# Percutaneous Pinning for Proximal Humerus Fractures: Insights From a Five-Year Retrospective Study

**DOI:** 10.7759/cureus.76600

**Published:** 2024-12-29

**Authors:** Abobaker Younis, Kenneth Kaar

**Affiliations:** 1 Orthopaedics and Traumatology, University Hospital Galway, Galway, IRL

**Keywords:** ireland, k-wires, minimally invasive fixation, percutaneous pinning, proximal humerus fractures

## Abstract

Introduction: Proximal humerus fractures are a common orthopedic challenge, particularly in older adults. Percutaneous pinning, a minimally invasive technique, aims to stabilize fractures while preserving soft tissue integrity. This study evaluates the functional and radiological outcomes of patients treated with percutaneous pinning at a single center over five years.

Methods: A retrospective review was conducted of patients who underwent percutaneous pinning for proximal humerus fractures between 2018 and 2023. Of 15 identified patients, 13 met the inclusion criteria of completing at least a three-month follow-up. Data were extracted from clinical records and radiological assessments, focusing on demographics, fracture characteristics, surgical details, complications, and functional outcomes.

Results: The study included 13 patients, of whom 12 (92%) were female, with a mean age of 60 years (range: 12-73 years). Most fractures (12/13, 92%) were three-part injuries resulting from low-energy trauma (11/13, 85%). All procedures were performed by a consultant shoulder specialist, using a combination of two to three K-wires with suture anchors. Anatomical alignment was achieved postoperatively in 10 (77%) cases, and radiological healing was observed in 10 (77%) by four weeks. At final follow-up, 12 (92%) patients demonstrated either excellent (9/13, 69%) or good alignment (3/13, 23%). Functionally, 11 (85%) patients reported either no pain (9/13, 69%) or mild pain (2/13, 15%). Similarly, 11 (85%) achieved either full functionality (8/13, 62%) or functionality with mild limitations (3/13, 23%) in arm use, with eight (62%) demonstrating a good range of motion. Complications were minimal, with no adverse events in nine (69%) cases. Early pin loosening occurred in three (23%) patients, and one (8%) required wire removal due to significant displacement. Follow-up duration ranged from three to 15 months (mean: 7.5 months), with patients attending an average of five clinic visits.

Conclusion: Percutaneous pinning provides reliable outcomes for selected proximal humerus fractures, yielding high rates of functional recovery and favorable radiological results. The use of suture anchors is particularly important for stabilizing the greater tuberosity and ensuring calcar support. With most patients experiencing satisfactory pain relief, functional use, and alignment, this minimally invasive approach demonstrates its efficacy and safety.

## Introduction

Proximal humerus fractures are among the most common fractures encountered in orthopedic practice, particularly in the elderly population [1]. They account for approximately 4-6% of all fractures, with the incidence rising in individuals over 65 years of age due to osteoporosis and low-energy trauma mechanisms such as falls from standing height [1,2]. These fractures significantly impact patients’ quality of life, often leading to pain, loss of function, and reduced independence.

Management of proximal humerus fractures depends on fracture type, patient age, comorbidities, and functional demands [3,4]. While non-displaced fractures are often managed conservatively, displaced and unstable fractures frequently require surgical intervention to restore alignment and ensure stability. Traditional surgical methods include open reduction and internal fixation (ORIF) and shoulder arthroplasty for complex fracture patterns [5,6]. However, these techniques are associated with increased surgical morbidity, longer recovery times, and higher complication rates in elderly or frail patients.

Minimally invasive techniques, such as percutaneous pinning, have gained attention for their ability to stabilize fractures while minimizing soft tissue disruption [7,8]. This approach involves the use of K-wires or threaded pins inserted through small skin incisions to achieve fracture reduction and stabilization. The advantages of this method include shorter operative time, reduced blood loss, preservation of soft tissue and vascular integrity, and lower infection rates [8,9]. Moreover, the addition of suture anchors to percutaneous pinning has been shown to enhance fixation by providing crucial calcar stability, particularly in valgus impacted fracture patterns [10]. This technique not only reduces the risk of pin migration but also plays a key role in stabilizing the greater tuberosity, ensuring optimal outcomes.

While percutaneous pinning is effective for many proximal humerus fractures, challenges remain, including the risk of pin migration, nonunion, and malunion, particularly in osteoporotic bone [7,11]. Patient selection and surgeon expertise are critical for optimizing outcomes, as the technique is best suited for two-part and certain three-part fractures [1,12]. Biomechanical studies suggest that multi-planar K-wire fixation provides superior stability compared to uniplanar techniques, further highlighting the importance of technique refinement [7].

Despite its advantages, there is a paucity of long-term data on the functional and radiological outcomes of percutaneous pinning. Comparative studies with traditional methods, such as locking plate osteosynthesis, suggest that minimally invasive approaches can yield similar outcomes while reducing surgical complications, particularly in elderly and osteoporotic patients [13,14].

This study aims to evaluate the outcomes of percutaneous pinning for proximal humerus fractures over a five-year period, focusing on radiological union, functional recovery, and complications. By contributing to the growing body of evidence supporting minimally invasive techniques, this work seeks to highlight the efficacy and safety of percutaneous pinning in managing proximal humerus fractures, particularly in carefully selected patient populations.

## Materials and methods

This is a retrospective observational study conducted at University Hospital Galway. The study analyzed cases of proximal humerus fractures treated with percutaneous pinning over a five-year period from 2018 to 2023. Ethical approval for the study was obtained from the hospital’s ethics committee (approval 546). All patient data were handled in compliance with confidentiality guidelines and data protection regulations, ensuring anonymity and adherence to ethical standards.

Patients were included if they were diagnosed with a proximal humerus fracture requiring surgical intervention, treated with percutaneous pinning using K-wires with or without suture anchors, and had a minimum follow-up of three months postoperatively [10]. Patients were excluded if they had incomplete medical records, follow-up less than three months, or fractures treated with other surgical methods, such as open reduction and internal fixation or arthroplasty.

All procedures were performed by an experienced consultant shoulder surgeon. Under general anesthesia and fluoroscopic guidance, closed reduction was achieved, followed by percutaneous insertion of two to four K-wires for fracture stabilization with suture anchored to calcar to augment fixation. Care was taken to ensure proper pin placement to minimize the risk of migration. Pins were typically removed after radiological evidence of fracture healing at follow-up.

Clinical and radiological data were extracted from medical records. Data collected included age, sex, mechanism of injury, fracture alignment, evidence of union, complications such as pin migration or malunion, pain status, range of motion, arm function as documented in clinic notes, and adverse events, including infections or reoperations.

The primary outcomes of the study were radiological union and functional recovery at the latest follow-up. Secondary outcomes included complication rates, such as pin migration, nonunion, and malunion. Descriptive statistics were used to summarize demographic and clinical data. Means and standard deviations were calculated for continuous variables, while categorical variables were expressed as percentages.

## Results

The study included 13 patients with an average age of 60 years (range 12 to 73; standard deviation 17). The cohort consisted of 92% females (12/13) and 8% males (1/13). All patients were referred to the shoulder specialist clinic within two weeks of injury. Fracture patterns included 8% two-part fractures (1/13) and 92% three-part fractures (12/13) (Table 1). The mechanism of injury was predominantly low-energy trauma (85%, 11/13), with high-energy trauma occurring in 15% (2/13), affecting one male and one young female (Table 1). Neurovascular status was intact in all cases (100%, 13/13). CT imaging was performed in 46% of patients (6/13) (Table 2).

**Table 1:** Patient Demographics and Fracture Characteristics This table summarizes the demographic characteristics and fracture patterns of the patient cohort, including age, gender distribution, fracture types, and the mechanisms of injury that led to the fractures.

Variable	Category	Frequency (N)	Percentage (%)
Age	Mean (SD)	60.46 (17.06)	
	Range	12-73	
Gender	Female	12	92%
	Male	1	8%
Mechanism of Injury	Low Energy	11	85%
	High Energy	2	15%
Fracture Type	2-part	1	8%
	3-part	12	92%

**Table 2:** Preoperative and Surgical Details This table details preoperative neurovascular status, the use of CT imaging, and the surgical techniques employed for fracture stabilization, including the proportion of patients receiving various methods of percutaneous pinning.

Variable	Category	Frequency (N)	Percentage (%)
Surgical Technique	Pinning Only	2	15%
	Wires + Anchors	11	85%
Neurovascular Status	Neurovascular Intact	13	100%
CT Imaging	Performed	6	46%
	Not Performed	7	54%
Fracture with Dislocation	Yes	1	8%
	No	12	92%

Surgical techniques varied; percutaneous pinning alone was performed in 15% of patients (2/13), while K-wires with suture anchors were used in 85% (11/13). The fracture and dislocation case underwent deltopectoral approach to reduce the humeral head (Table 2). Postoperatively, immediate alignment was anatomical in 77% (10/13), with negligible displacement observed in 23% (3/13). Wires were removed around three to four weeks in 77% of cases (10/13), and physiotherapy commenced at four weeks in 77% of cases (10/13) (Table 3).

**Table 3:** Immediate Postoperative and Healing Outcomes This table highlights the immediate postoperative alignment, timing of wire removal, and fracture healing outcomes within the first four weeks after surgery.

Variable	Category	Frequency (N)	Percentage (%)
Immediate Postop Alignment	Anatomical	10	77%
	Negligible Displacement	3	23%
Wire Removal Time	3-4 weeks	10	77%
Healing at 4 weeks	Good Healing and alignment	10	77%
	Minimally Displaced	1	8%
	Significantly Displaced	2	15%

At the latest follow-up, fracture healing was rated as good in 77% (10/13), minimally displaced in 8% (1/13), and significantly displaced in 15% (2/13) (Table 3). Final alignment was excellent in 69% (9/13), satisfactory in 15% (2/13), and unsatisfactory in 8% (1/13), with one case (8%) not assessed (Table 4). Imaging revealed no complications in 69% (9/13), early pin loosening in 23% (3/13), and significant displacement requiring wire removal in 8% (1/13).

**Table 4:** Final Alignment and Functional Outcomes This table outlines the final alignment of fractures, pain levels, range of motion, and functional use of the affected arm as assessed at the latest follow-up.

Variable	Category	Frequency (N)	Percentage (%)
Final Alignment	Excellent	9	69%
	Satisfactory	2	15%
	Unsatisfactory	1	8%
	Not Assessed	1	8%
Pain Status	No Pain	9	69%
	Mild Pain	2	15%
	Moderate Pain	1	8%
	Missing	1	8%
Range of Motion	Good	8	62%
	Moderate Restriction	3	23%
	Severe Restriction	1	8%
	Missing	1	8%
Functional Arm Use	Fully Functional	9	69%
	Functional with Limitations	2	15%
	Limited	1	8%
	Missing	1	8%

Pain outcomes showed 69% of patients (9/13) reported no pain, 15% (2/13) had mild pain, and 8% (1/13) reported moderate pain, while data were missing for 8% (1/13) (Table 4). Range of motion was good in 62% (8/13), moderately restricted in 23% (3/13), and severely restricted in 8% (1/13), with data missing in 8% (1/13). Functional arm use was fully functional in 69% (9/13), functional with limitations in 15% (2/13), and limited in 8% (1/13), with 8% (1/13) missing (Table 4). Complications reported included no issues in 69% (9/13), fracture displacement in 8% (1/13), pin site infection requiring pin removal in 8% (1/13), and pin loosening in 15% (2/13), with no medial pin migration reported (Table 5).

**Table 5:** Complications and Follow-up This table presents the complications encountered during the study, including pin site issues and fracture displacement, along with the duration of follow-up and the number of clinic visits required by patients.

Variable	Category	Frequency (N)	Percentage (%)
Complications Reported	No Complications	9	69%
	Fracture Displacement	1	8%
	Pin Site Infection	1	8%
	Pin Loosening	2	15%
Final Assessment	Discharged Satisfactorily	11	85%
	ORIF Required	1	8%
	Reverse TSR Offered	1	8%
Follow-up Duration	Mean (SD)	7.5 (3.5)	
	Range	3-15 months	
Number of Visits	Mean (SD)	5 (1.08)	
	Range	4-7 visits	

Follow-up duration ranged from three to 15 months, with an average of 7.5 months. Patients attended between four and seven clinic visits, with a mean of five visits (Table 5).

## Discussion

The effectiveness of percutaneous pinning with K-wires, with or without suture anchors, as a treatment for proximal humerus fractures has been demonstrated in this study. The majority of patients achieved satisfactory fracture healing and functional recovery. Anatomical alignment was achieved in most cases, and a significant proportion of patients demonstrated fully functional arm use at the latest follow-up. Pain outcomes were favorable, with most patients reporting no or minimal discomfort. Complication rates were low, with only a few cases showing pin loosening or displacement. Importantly, no major neurovascular injuries were observed, supporting the safety of this minimally invasive approach.

These findings are consistent with prior research that has evaluated the role of percutaneous pinning in managing proximal humerus fractures. Alexa et al. reported similar success rates in achieving fracture healing and maintaining anatomical alignment, particularly for two- and three-part fractures [1]. Baig et al. also found comparable outcomes using this technique, particularly in cases of three-part fractures [10]. Comparable results were reported by Fenichel et al., who emphasized the advantages of percutaneous pinning in reducing soft tissue disruption [8]. Studies by Angehrn and Schultheiss highlighted the role of percutaneous wire fixation as an early and effective treatment option for proximal humerus fractures, demonstrating its ability to achieve good results with minimal invasiveness [15].

The preservation of neurovascular integrity observed in this study aligns with findings reported by Seyhan et al. and Dinget al., further reinforcing the safety of this technique [9,12]. Jiang et al. also demonstrated that percutaneous pinning offers biomechanical stability and can achieve favorable outcomes in proximal humerus fractures [16]. These biomechanical benefits were further supported by Naidu et al., who emphasized the role of pin placement in achieving fixation stability and preventing migration [17].

The functional outcomes observed in this study reflect those reported by Muncibi et al. and Ortmaier et al., where good arm function with minimal complications was achieved following percutaneous pinning [13]. In the current cohort, most patients regained full functional use of their arm, which is consistent with outcomes in elderly patients with three-part fractures [14,18]. Rowles and McGrory demonstrated that anatomic placement of pins is essential for achieving favorable outcomes and avoiding complications such as pin migration or soft tissue irritation [19]. This further supports the role of percutaneous pinning as a reliable option for restoring functional independence, particularly in patients with lower-energy trauma.

In terms of complications in this study, pin loosening and displacement were minor and align with those reported in previous studies. El-Alfy documented similar rates of pin-related complications, though they were infrequent when proper surgical techniques were applied [11]. Similarly, Jayarajah et al. and Soete et al. noted that pin loosening and infections remained uncommon with careful technique and monitoring [20,21]. Gunst et al. further highlighted the importance of proper pin placement and noted favorable results using minimally invasive techniques for valgus displaced fractures [22].

The advantages of percutaneous pinning observed in this study are well-documented in the literature. Carbone et al. and Esen et al. demonstrated that this technique is associated with shorter operative times, minimal soft tissue disruption, and early mobilization compared to open reduction and internal fixation [6,7]. Jiang et al. also reported favorable results with minimally invasive plate osteosynthesis, further supporting the role of minimally invasive techniques in managing proximal humerus fractures [23]. Furthermore, Baig et al. suggested that the use of suture anchors alongside K-wires may provide additional stability, which may explain the favorable outcomes observed in this cohort [10].

The findings of this study further emphasize that percutaneous pinning remains a valuable treatment option for two- and three-part valgus impacted proximal humerus fractures, particularly in elderly or frail patients. Proper patient selection remains critical to achieving favorable results, as highlighted by Seyhan et al. and Baig et al. [10,12]. Laflamme et al. emphasized that technical factors, such as pin trajectory and placement, significantly affect the stability of fixation and ultimate outcomes [24]. Fractures that are amenable to closed reduction and patients with lower functional demands are more likely to benefit from this approach while minimizing complications.

Despite its advantages, this study is not without limitations. The small sample size, stemming from stringent inclusion criteria and the predominance of low-demand elderly patients treated conservatively at our institution, limits the generalizability of the findings. The retrospective, single-center design introduces potential selection bias and restricts the broader applicability of the results. Varying follow-up durations further complicate the assessment of long-term outcomes and functional recovery. Additionally, the reliance on clinical documentation, rather than standardized patient-reported outcome measures (PROMs), may have introduced variability in evaluating functional outcomes. The observed complications, including pin loosening and significant displacement in a small number of cases, underscore the need for refinement in surgical techniques and enhanced postoperative monitoring.

Further research is needed to confirm the long-term efficacy and safety of percutaneous pinning with K-wires and suture anchors. Prospective studies with larger sample sizes and standardized follow-up protocols are essential. Biomechanical studies exploring improvements in pin placement techniques and strategies to minimize pin loosening would also contribute to refining this approach.

## Conclusions

This study demonstrates that percutaneous pinning with K-wires, augmented with suture anchors when necessary, is a safe and effective option for managing two- and three-part valgus impacted proximal humerus fractures. The majority of patients achieved reliable fracture healing, good functional recovery, and minimal complications. The technique preserves soft tissue integrity and offers favorable outcomes, particularly in elderly patients with low-energy fractures where open techniques may present higher risks. With proper patient selection and ongoing refinement of surgical techniques, percutaneous pinning remains a valuable treatment option for proximal humerus fractures.
